# The Correlation Between Immune Invasion and SARS-COV-2 Entry Protein ADAM17 in Cancer Patients by Bioinformatic Analysis

**DOI:** 10.3389/fimmu.2022.923516

**Published:** 2022-06-03

**Authors:** Kai Wang, Haoyue Deng, Binghui Song, Jiayue He, Shuguang Liu, Jiewen Fu, Lianmei Zhang, Dabing Li, Kyathegowdanadoddi Srinivasa Balaji, Zhiqiang Mei, Jingliang Cheng, Junjiang Fu

**Affiliations:** ^1^ Key Laboratory of Epigenetics and Oncology, Research Center for Preclinical Medicine, Southwest Medical University, Luzhou, China; ^2^ Department of Pathology, The Affiliated Huaian No. 1 People’s Hospital of Nanjing Medical University, Huai’an, China; ^3^ Basic Medical School, Southwest Medical University, Luzhou, China; ^4^ PG Department of Biotechnology, Teresian College, University of Mysore, Mysore, India

**Keywords:** ADAM17, cancer, SARS-CoV-2, susceptibility, therapeutics, small molecule

## Abstract

SARS-Cov-2 caused the COVID-19 pandemic worldwide. ADAM17 functions as a disintegrin and transmembrane metalloproteinase domain protein involved in the regulation of SARS-CoV-2 receptor ACE2. However, its impact on cancer patients infected with COVID-19 and its correlation with immune cell infiltration is unclear. This study compared ADAM17 expression between normal and tumor tissues based on GEPIA. The correlations between ADAM17 expression and immune cell infiltration and immunomodulators were investigated. Besides, treated drugs for targeting ADAM17 were searched in the TISDB database. We found that ADAM17 was highly conserved in many species and was mainly expressed in lung, brain, female tissues, bone marrow and lymphoid tissues. It was also highly expressed in respiratory epithelial cells of rhinitis and bronchus. ADAM17 expression in tumors was higher than that in several paired normal tissues and was negatively correlated with the prognosis of patients with malignant tumors. Interestingly, ADAM17 expression significantly correlated with immunomodulators and immune cell infiltration in normal and tumor tissues. Moreover, eight small molecules targeting ADAM17 only demonstrate therapeutic significance. These findings imply important implications for ADAM17 in cancer patients infected with COVID-19 and provide new clues for development strategy of anti-COVID-19.

## Introduction

SARS-Cov-2 has caused a worldwide pandemic of Corona Virus Disease 2019 (COVID-19) since December 2019. It is an enveloped virus belonging to the beta coronavirus family that has infected about 300 million people worldwide and killed more than 5 million. The process by which the entry of SARS-Cov-2 into host cells is mediated by transmembrane spike (S) glycoproteins (1). Envelope-anchored S protein is cleaved into functional S1 and S2 subunits in the boundary region. S1 binds to host cell receptors, and S2 fuses with coronavirus membranes and cell membranes (2). Emerging evidence suggests that both S1 protein-receptor interaction and the fusion of the viral envelope with the host cell membrane are critical two steps in driving a successful infection of SARS-Cov-2 (3).

Recently, multiple potential SARS-Cov-2-related receptors have been identified, including heparan sulfate proteoglycans (HSPGs), angiotensin-Converting Enzyme 2 (ACE2), aminopeptidase N (APN), cathepsin L (CTSL), Heat Shock Protein A5 (HSPA5), transmembrane serine protease 2 (TMPRSS2), furin, O-Acetylated Sialic Acid (O-Ac-Sia) (4–6). Among them, ACE2 serves as a functional receptor of SARS-Cov-2 spike glycoprotein that is widely expressed on the membrane of apical epithelial cells, especially on ciliated cells (7). In addition, ACE2 is also found in kidney, cardiovascular, and gastrointestinal tissues. It can bind to the B domain of COVID-19 virus S protein, enabling the virus to enter target cells and start replicating leading to infection (8). ADAM metallopeptidase domain 17 (ADAM17, Ensembl: ENSG00000151694; OMIM: 603639), also known as ADAM18, CD156B, CSVP, NISBD, NISBD1 and TACE, is another newly identified potential receptor for SARS-Cov-2 (9). As a type-I multidomain transmembrane protein, the pro-domain of ADAM17 possesses catalytic activity (10). Once the pre-domain is hydrolyzed, ADAM17 participates in the hydrolysis and abscission of various extracellular functional regions of proteins, such as ACE2 (11). Increased ACE2 shedding triggers enhanced viral infection.

Infection of SARS-Cov-2 is closely connected with regulating the body’s cellular immunity and humoral immunity. Initially, the body protects against viral infection through humoral immunity, i.e., neutralizing antibody (NAb) levels (12). Within two weeks of infection, plasma cells increase and virus-specific IgM and IgG can be detected (12). In the advanced stage of the disease, lymphocytes, CD4+T cells, CD8+T cells and natural killer cells (NK) are reduced in COVID-19 patients, accompanied by cytokine storms. In addition, PD-1, T-cell immunoglobulins, and mucin domain-3 (Tim-3) have also contributed to exacerbating infection (13), indicating the occurrence of adaptive immune escape.

Increasing evidence indicates that malignant tumors can induce the transport of immature neutrophils and monocytes into the tumor microenvironment, thereby causing immunosuppression (14). Besides, after treatment with chemotherapeutic agents and immunosuppressants, the body’s immunity will also decline sharply, making the immune system of cancer patients abnormally changed and patients more susceptible to infection. Immune cell infiltration in the tumor microenvironment plays a key role in tumor development and influences cancer patients’ prognosis. Studies have shown that patients with advanced cancer are more likely to infect with COVID-19 and have a worse prognosis than normal individuals (15, 16).

ADAM17 has been reported to induce local tumor invasion and metastasis *via* degrading the cell basement membrane and extracellular matrix and affecting tissue remodeling. ADAM17 is usually expressed in various malignant tumors, while rarely expressed in normal cells (17), implying its specificity in the tumor. The effect of ADAM17 on cancer patients infected with COVID-19 and its correlation with immune cell infiltration have not been fully elucidated. This study investigated ADAM17 expression in cancer patients and/or normal individuals by bioinformatics analysis.

## Materials and Methods

### Databases and Sources

The expressions of ADAM17 (Ensembl ID: ENSG00000151694) in mRNA and protein from normal human tissues were obtained from the database of Human Protein Atlas (HPA) (https://www.proteinatlas.org/ENSG00000151694-ADAM17) (18, 19). Immunohistochemical or immunofluorescence images of ADAM17 in normal tissues (v20.proteinatlas.org/ENSG00000151694-ADAM17/tissue), tumor tissues (v20.proteinatlas.org/ENSG00000151694-ADAM17/pathology), and cancer cell lines (v20.proteinatlas.org/ENSG00000151694-ADAM17/cell#img) were obtained from the HPA database (20, 21). The ADAM17 expressions were verified using the project of Genotype-Tissue Expression (GTEx). The Gene Expression Profiling Interactive Analysis (GEPIA) dataset (GEPIA 2) was obtained from the website (http://gepia2.cancer-pku.cn/#index) (22) to compare the expressions between tumor and matched normal tissues. The ADAM17 structure with indicated residues of amino acids for each domain was gained in the Uniprot database (https://www.uniprot.org/uniprot/P78536) (UniProtKB/Swiss-Prot number P78536.1). The NCBI (National Center for Biotechnology Information) database was applied to perform homology analysis (https://www.ncbi.nlm.nih.gov/homologene/2395) (23). The correlation between ADAM17 and treated drugs targeting ADAM17 was performed in the TISDB database (http://cis.hku.hk/TISIDB/browse.php?gene=ADAM17) (24).

### HPA (Human Protein Atlas) Analysis for ADAM17

The mRNA and protein expressions of ADAM17 were analyzed in normal and tumor tissues from the HPA database (https://www.proteinatlas.org/ENSG00000151694-ADAM17) (21). ADAM17 mRNA levels in various normal tissues were gained from the consensus datasets of three sources of HPA, GTEx, and FANTOM5 (18, 25). Consensus Normalized expression levels for tissues and blood cells were acquired through the above three datasets (v20.proteinatlas.org/about/assays+annotation#normalization_rna). For IHC staining in these data, two antibodies for ADAM17 (cat #: CAB025906, R&D Systems; cat #: HPA010738, Sigma-Aldrich) were used for tissue and cell line IHC staining (26).

### GEPIA and Prognostic Value Analysis for ADAM17

The ADAM17 mRNA expressions in 8,587 normal samples and 9,736 tumor tissues were analyzed with GEPIA (22). The correlation between ADAM17 expression level and median overall survival (OS) was also analyzed using the GEPIA database.

### Immunohistochemistry (IHC)

ADAM17 antibody for IHC was purchased from Sigma (HPA010738, Sigma-Aldrich). The IHC protocols were described previously (27–29). β-actin was served as an internal control.

### Correlation Analysis for ADAM17 Expression and Immunoregulation

We downloaded the pan-cancer dataset (PANCAN, N=10535, G=60499) from the UCSC database (http://xenabrowser.net/) and extracted the ADAM17 gene and 60 immune checkpoint regulatory genes (30), 150 immune regulatory genes (41 chemokines, 18 receptors, 21 MHCs, 24 immunoinhibitors, 46 immunostimulators) and 64 tumor-related immune cells for immune checkpoint genetic analysis, immunomodulatory gene analysis, immune cell analysis, and immune invasion analysis, respectively. Log2 (x+0.001) transformation was performed for each expressed value. For immune cell infiltration analysis, Pearson correlations of these genes were calculated using TIMER (31) and deconvo xCell (32) algorithms of the R software package IOBR (version 0.99.9) (33).

### Statistical Analysis

ADAM17 expressions of samples in survival analysis were divided into two groups (high vs. low) using a median expression with overall survival (OS). Logrank with *P* < 0.05 was considered a significant difference.

## Results

### Highly Conserved ADAM17 and Its Structure for Ectodomain Shedding

**Figure 1:**
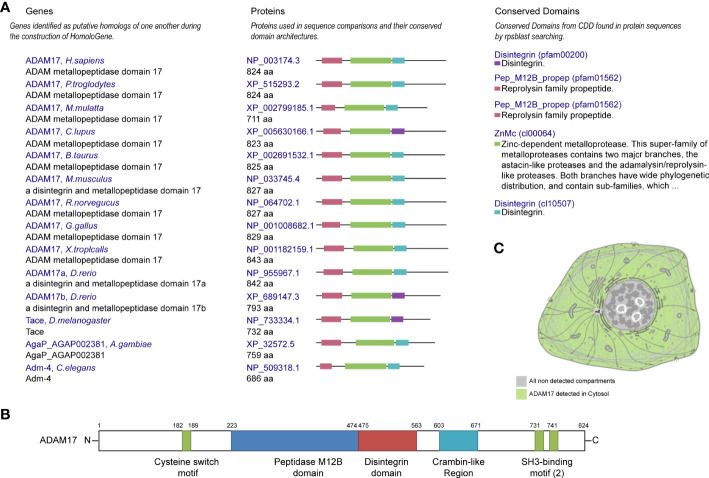
Conservation, structure, and cellular localization of ADAM17 in humans. **(A)** Conservation for ADAM17 in the indicated different species. **(B)** Structures of ADAM17. **(C)** A diagrammatic sketch of ADAM17 cellular localization. Green indicates ADAM17 in the cytosol, whereas gray indicates the absence.

Protein homology analysis suggested that ADAM17 protein is highly conserved in various species, including *H. sapiens*, chimpanzee, rhesus monkey, dog, cow, mouse, rat, chicken, zebrafish, fruit fly, mosquito, *C. elegans*, and frog (**Figure 1A**), suggesting that ADAM17 has the potential to recognize SARS-CoV-2 spike proteins, which is similar to the function of ACE2 (23, 34). The UniProtKB/Swiss-Prot database analysis showed that ADAM17 has a peptidase M12B domain, a disintegrin domain, a crambin-like region, a cysteine switch motif and two SH3-binding motifs (**Figure 1B**). The longest domain (252 aa) of peptidase M12B (M12B proteinases, adamalysins or reprolysins) is a catalytic domain with the metal endopeptidase activity (35, 36), which is zinc ion-dependent endopeptidases. The conserved cysteine presents in the cysteine-switch motif and binds with the catalytic zinc ion to inhibit the enzymatic activity, whereas cysteine dissociates from the zinc ion upon activation of the peptide to release the activity of the enzyme (37). Disintegrins, a family of small proteins in viper venoms, initially act as potent inhibitors of platelet aggregation and integrin-dependent cell adhesion (38), thus participating in the cellular-extracellular matrix and subcellular interactions. The SH3-binding motif originally binds to the Src homology 3 (SH3) region, the latter of which binds with target proteins and mediates the functional assembly of protein complexes. The crambin-like domain is an amphipathic region (39). In addition, the topological extracellular domain, the helical transmembrane, the topological cytoplasmic domain and signal peptide are located at 215-671, 672-692, 693-824, and 1-17 amino acid residues, respectively. ADAM17 locates in the cytoplasm of cells (**Figure 1C**, and **Supplementary Figures 1A–D**) and the cytoplasm and membrane of tissues (**Supplementary Figure 1E**). Collectively, these results suggest that ADAM17 may play a role in proteolysis and extracellular domain shedding of diverse proteins, including ACE2 (40).

### ADAM17 Is Mainly Expressed in Placental, Lung, Nasopharynx, Bronchus, Bone Marrow and Lymphatic Tissues

**Figure 2:**
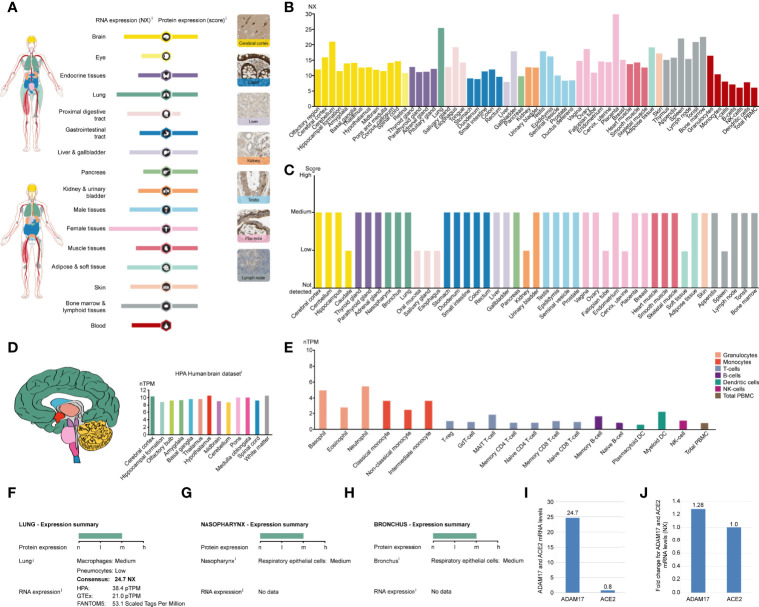
ADAM17 expression in normal tissues. **(A)** The summary of ADAM17 mRNA and protein expressions. Color-coding columns are based on tissue groups, and each consists of tissues with common functional features. Respective images are the immunohistochemistry (IHC) staining of ADAM17 from the HPA in normal tissues. **(B)** ADAM17 mRNA expressions from normal tissues. An NX value of 1.0 is defined as a threshold for ADAM17 mRNA expression. NX, normalized expression. **(C)** ADAM17 protein levels in normal tissues were from the HPA. Protein expression data are presented for every 44 tissues. **(D)** Brain mRNA expression for ADAM17. **(E)** Immune cell type expression for ADAM17 mRNA. The expression values were calculated as nTPM, which resulted from the internal normalization pipeline for 18 immune cell types and total PBMC (peripheral blood mononuclear cells). HPA, Human Protein Atlas. **(F)** Expression summary in the lungs. pTPM, protein-transcripts per million. **(G)** Expression summary in the nasopharynx. **(H)** Expression summary in the bronchus. **(I)** Comparison mRNA expressions for ADAM17 and ACE2 in the lungs (NX values) from the HPA dataset. **(J)** The mRNA expression comparison between ADAM17 and ACE2 in normal heart tissues.

ADAM17 mRNA has low specificity in various human tissues and is highly expressed in the placenta (NX: 29.0), followed by the lung (NX: 24.7), and yet extremely low in total peripheral blood mononuclear cell (PBMC) (NX: 6), liver tissue (NX: 7.8) and NK cells (NX: 6) (**Figures 2A, B**). ADAM17 protein has cytoplasmic and membranous expression in various tissues, with moderate expression in 36 tissue types and low expression in the remaining 9 tissues (**Figures 2A, C**). Brain mRNA expression for ADAM17 showed either low tissue specificity or low region specificity (**Figure 2D**). Immune cell type expression for ADAM17 mRNA showed low immune cell specificity but higher in monocytes and granulocytes (**Figure 2E**).

Since the process by which SARS-Cov-2 entering host cells is mediated by transmembrane spike (S) glycoproteins. ACE2 serves as one of the main receptors for SARS-COV-2 that can bind to the B domain of the COVID-19 viral S protein, which in turn allows the virus to enter the target cells and begin to replicate leading to infection. However, ACE2 is found to be widely expressed on the membrane of the pulmonary ciliated epithelium (7). Besides, in an *in vivo* study using an ACE2 mutant mouse model, it was found that acute lung injury and even lung failure were aggravated by injection of coronavirus spike, while treatment with recombinant ACE2 significantly reduced acute lung failure (41). In this regard, it has been speculated that increased plasma membrane binding to ACE2 may lead to higher infections (42). Here, we investigated the role of ADAM17 in the human respiratory tract and found that its mRNA or protein levels were markedly elevated in lung tissue, rhinitis and bronchus (**Figures 2B, C**, **F–H**). In-depth analysis showed that ADAM17 is expressed in pneumocytes (31.67%), endothelial cells (28.33%), macrophages (10%) and bronchial epithelial cells (5%) (**Table 1**). Immunohistochemical staining revealed that the expression of ADAM17 protein was detected in alveolar macrophages (blue arrows)/type I (red arrows) and type II (black arrow) alveolar epithelial cells, which are mostly located in the cytoplasm/cytoplasm (**Supplementary Figures 2A, B**). Furthermore, HPA, GTEx and FANTOM5 database analysis suggested that the mRNA level of ADAM17 in lung tissue was significantly higher than ACE2 (30.88 = 24.7/0.8, **Figure 2I**). As ADAM17 is a type I multi-domain transmembrane protein, and participates in the hydrolysis and shedding of ACE2, the latter of which further aggravates viral infection. We suggest that ADAM17 might also play a key role in entering SARS-COV-2 into the lung cells.

**Table 1:** ADAM17 RNA expression in different cells from the lung.

Cell types	Percentages
Pneumocytes	31.67
Bronchial epithelium	5
Endothelial cells	28.33
Macrophages	10
Other cell types	25

data was normalized to nine samples from HPA RNA-sequencing.

While attacking the new coronavirus in the lungs, many cytokines affect organs outside the lungs, such as the heart, kidney, liver, and other organs. Expression of ADAM17 in those tissues was also conducted in this study. The expression of ADAM17 in normal and tumor tissues was lower in the kidney and liver than in the heart (**Figures 2A, B**). Thus, we focused on analyzing the expression of ADAM17 in normal heart tissue because the cytokines can easily increase the severity in younger people severity and eventually lead to death. The results are shown in **Supplementary Figure 3**. The expression of ADAM17 protein in normal heart tissue was medium in both two antibodies (HPA010738, CAB025906) (**Supplementary Figures 3A, B**, https://www.proteinatlas.org/ENSG00000151694-ADAM17/tissue/heart+muscle), whereas the expression in normal heart tissue of ACE2 protein was low in the ACE2 antibody CAB080024, and antibody CAB080025, but there were no signals detected in the ACE2 antibody HPA000288, antibody CAB026174, and antibody CAB026213 (data not shown, URL: v20.proteinatlas.org/ENSG00000130234-ACE2/tissue/heart+muscle). Both proteins of ADAM17 and ACE2 were also found to be the membranous and cytoplasmic expression, mainly in the myocytes (**Supplementary Figures 3A–D**, data not shown). The mRNA expressions comparison between ADAM17 and ACE2 in heart tissues showed that the ADAM17 mRNA levels are 1.28-fold higher than ACE2 (**Figure 2J**).

### Differential Expression of ADAM17 in Tumor Tissues and Corresponding Normal Tissues

**Figure 3:**
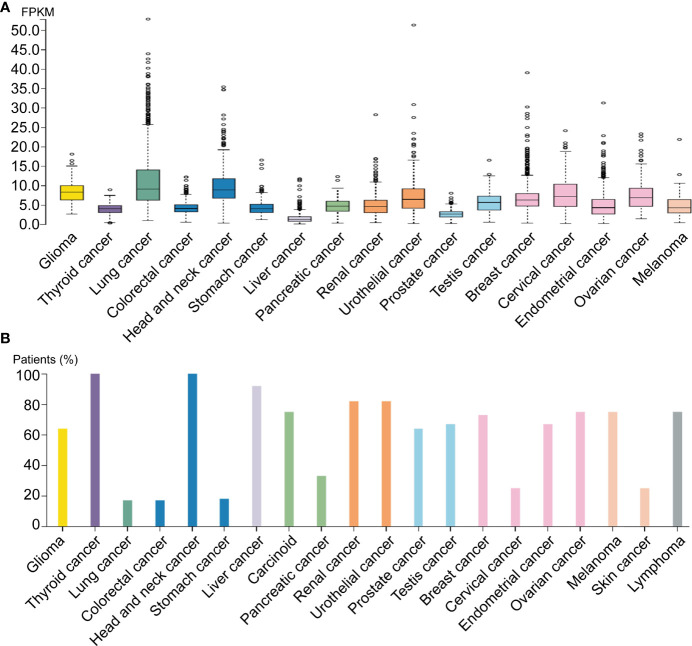
ADAM17 expression levels in different human cancers. **(A)** ADAM17 mRNA expression in different cancer tissues. **(B)** The protein expressions of ADAM17. In each cancer type, color-coded bars show the percentage of patients with high and medium levels of ADAM17 protein. The cancer types are color-coded according to organ type, and cancer originates.

**Figure 4:**
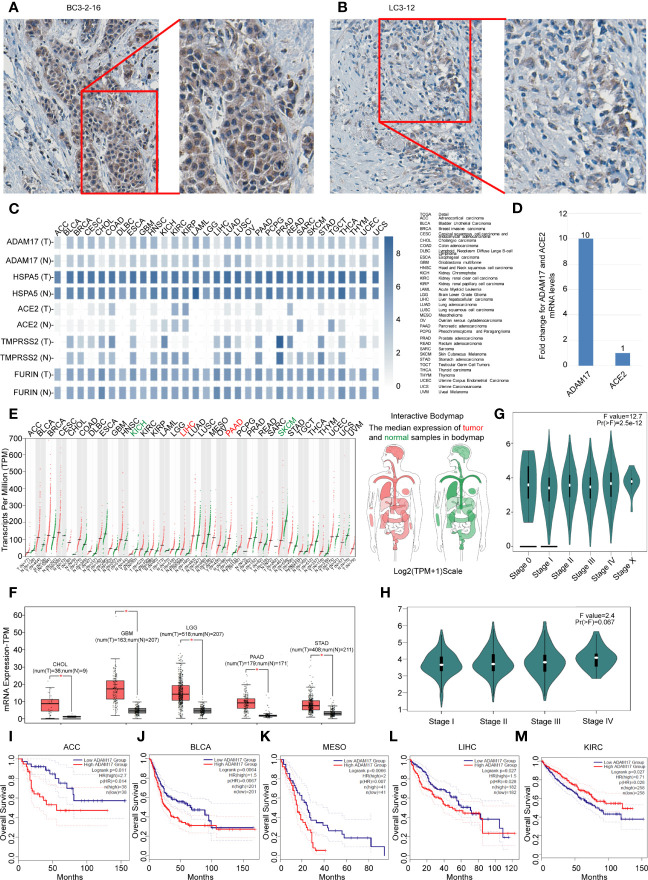
Differential expressions of ADAM17, HSPA5, ACE2, FURIN, and TMPRSS2 in tumors and corresponding normal tissues, and prognostic values of ADAM17 expression in multiple cancer types. **(A)** The representative IHC images of tumors from Chinese breast cancer patients. **(B)** The representative IHC images of tumors from Chinese lung cancer patients. IHC was performed by ADAM17 antibody (HPA010738, Sigma-Aldrich). B&D, enlarged images from A&C respectively. **(C)** Expression comparisons of ADAM17 with HSPA5, TMPRSS2, FURIN, and ACE2 in 31 types of normal and cancer tissues. “T” stands for tumors, whereas “N” denotes matched normal tissues. The cancer types of full names are shown on the right panel. **(D)** mRNA comparison between ADAM17 and ACE2 in lung cancer from the TCGA dataset. **(E)** The ADAM17 expression profiles across all cancers and paired normal individuals of tissues with dot plots. **(F)** ADAM17 was overexpressed in 5 cancer types with box plots. ADAM17 mRNA levels in cancers and matched normal individuals of tissues were obtained through the dataset of GEPIA. *P<0.05. **(G)** Pathological stage plots in pan-cancer. Pr(>F), 2.5e-12. **(H)** Pathological stage plots in LUAD. Pr(>F), 0.067. **(I-M)** The prognostic values for ADAM17 in five cancer types from the GEPIA dataset. I-M indicates ACC, BLCA, MESO, LIHC, and KIRC. *P < 0.05. The cancer types of full names are presented on the right panel of **Figure 4C**. HR, Hazards Ratio. GEPIA, Gene Expression Profiling Interactive Analysis.

By analyzing distinct malignant tissues, we found that ADAM17 was highly expressed in lung cancer tissues (9 FPKM) and low in liver cancer tissues (1.3 FPKM) (**Figure 3A**). ADAM17 protein is located in the membrane and cytoplasm in distinct tumor tissues. Moreover, weak to moderate cytoplasmic immune responses were observed in most tumor tissues (**Figures 3B**, **4A, B**, data not shown).

Next, we analyzed the expression values of ADAM17, HSPA5, TMPRSS2, FURIN, and ACE2 in different cancers and corresponding normal individuals. As shown in **Figure 4C**, compared with relative normal tissues, only ADAM17 was highly expressed in lung cancer, while the other four genes did not show any significant difference. ADAM17 mRNA levels were lower than HSPA5 and FURIN but higher than ACE2 and TMPRSS2 in most cancer types (**Figure 4D**). Furthermore, we analyzed ADAM17 mRNA and ACE2 mRNA from 994 samples through the TCGA dataset and found that ADAM17 mRNA level was 10-fold higher than ACE2 (**Figure 4D**), suggesting that ADAM17 might play a critical role in SARS-Cov-2 entry in patients of the lungs, which was supported partially by a systematic review that showed the level of malignancy in lung cancer patients correlated with the likelihood of having COVID-19 (43).

Then, the ADAM17 mRNA expression profile across distinct tumors and the corresponding normal tissues in pan-cancer were assessed with the GEPIA dataset (**Figures 4D, E**). We found that the ADAM17 gene was expressed in nearly all tumor tissues, and was significantly upregulated in five types of tumors, including cholangio carcinoma (CHOL), brain lower-grade glioma (LGG), glioblastoma multiforme (GBM), pancreatic adenocarcinoma (PAAD), and stomach adenocarcinoma (STAD) (**Figure 4F**), and these tumor tissues expressing ADAM17 at higher levels than corresponding normal tissues in the brain and digestive tract (**Figure 4E**). However, no significant difference in ADAM17 expression was observed in the remaining tumor tissues (**Figure 4E**). The gene expression of ADAM17 in human tumors and matched normal tissues were validated in the database of ONCOMINE (Data not shown). ADAM17 expressions in lung cancer were increased, but not significantly compared to normal tissues in the TCGA dataset (Data not shown).

Next, the pathological stage plots in all indicated 26 cancer types (http://gepia2.cancer-pku.cn/#analysis) were performed and the results showed that the ADAM17 expressions are positively correlated with advanced tumor stages of the pan-cancer [Pr(>F), 2.5e-12] (**Figure 4G**); specific for kidney chromophobe (KICH) [Pr(>F), 0.022], liver hepatocellular carcinoma (LIHC) [Pr(>F), 0.0106], and ovarian serous cystadenocarcinoma (OV) [Pr(>F), 0.0139]; but not for lung cancers, either lung adenocarcinoma (LUAD) [Pr(>F), 0.067] (**Figure 4H**) or Lung squamous cell carcinoma (LUSC) [Pr(>F), 0.945] (Data not shown).

The prognostic value for ADAM17 in pan-cancer was investigated by Kaplan-Meier analysis and found that the high expression of ADAM17 significantly decreased patients’ overall survival (OS) in four types of cancers, i.e., adrenocortical carcinoma (ACC), breast invasive carcinoma (BLCA), mesothelioma (MESO), and liver hepatocellular carcinoma (LIHC) (**Figures 4I–L**); whereas low expression of ADAM17 significantly decreased patient OS only in kidney renal clear cell carcinoma (KIRC) (**Figure 4M**). These results indicate that the OS of cancer patients is markedly reduced in most types of malignant tumors.

### ADAM17 Expression Is Associated With Immune Cell Infiltration in Several Tumors and Is a Potential Drug Target

**Figure 5:**
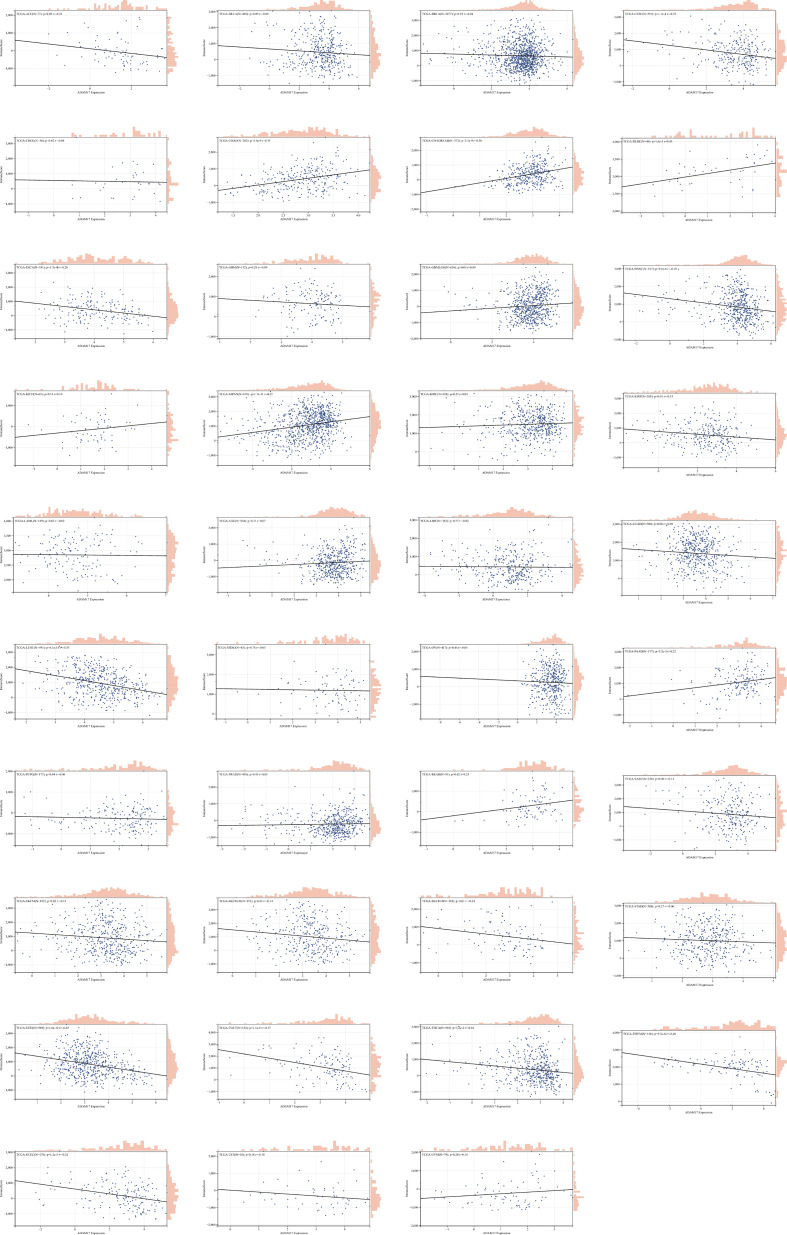
The correlation between ADAM17 expression and immune invasion score in multiple cancer types.

Due to the indispensability of the immune system during antiviral processes, the correlations between the expression of ADAM17 and the level of immune infiltration in cancers were performed. By analyzing the correlation between the ADAM17 gene and immune invasion scores in 9,555 tumor samples from 39 cancer species, we observed a significant correlation between the expression of this gene and immune invasion in 21 cancer species, with 7 (GBMLGG, COAD, COADREAD, KIPAN, READ, PAAD, DLBC) positive and 14 (CESC, ESCA, STES, KIRP, UCEC, HNSC, LUSC, THYM, THCA, SKCM-M, SKCM, TGCT, SKCM-P, ACC) negative correlations (**Figure 5**).

**Figure 6:**
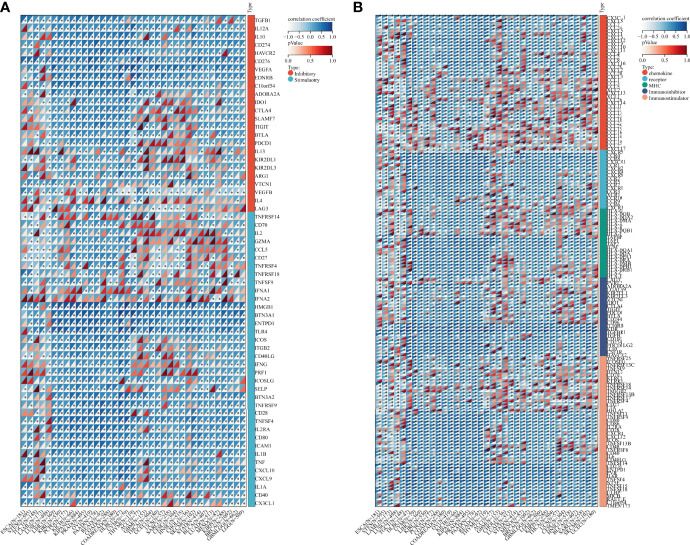
Bioinformatics analysis of the immunomodulatory role of ADAM17 in multiple cancer types. **(A)** Correlation between ADAM17 and 60 genes related to immune checkpoints. **(B)** Correlation between ADAM17 and 150 immunomodulators (41 chemokines, 18 receptors, 21 MHCs, 24 immunoinhibitors, and 46 immunostimulators). *P <0.05.

Besides, we collected ADAM17 gene and 60 immune checkpoint regulatory genes, 150 immune regulatory genes (41 chemokines, 18 receptors, 21 MHCs, 24 immunoinhibitors, 46 immunostimulators) and 64 tumor-related immune cells for immune checkpoint genetic analysis, immunomodulatory gene analysis, immune cell analysis, and immune invasion analysis, respectively. Bioinformatics analysis aimed to explore the importance of the ADMA17 immune response in identifying different types of cancer that might benefit from anti-ADAM17 therapy. Results showed that the expression of ADAM17 was mutually exclusive of several tumor immune checkpoints, like CD40LG, CX3CL1, VEGFB, TNFSF9, IFNA2 in ESCA, STES, LAML, DLBC, UVM, THYM, KIRP, LGG or GBMLGG (**Figure 6A**). Furthermore, ADAM17 was negatively connected with several immunoregulatory genes (i.e., CCL25, CCL15, TNFRSF18, HHLA2, CXCL12, CXCL17, TMIGD2, CXCR5, TNFRSF9, CCL25, CXCL17, IAG3, TNFRSF4) in ESCA, STES, LAML, COAD, KIPAN, PRAD, READ, THYM, LUAD, MESO, GBMLGG, THCA, UCS, or HNSC (**Figure 6B**).

**Figure 7:**
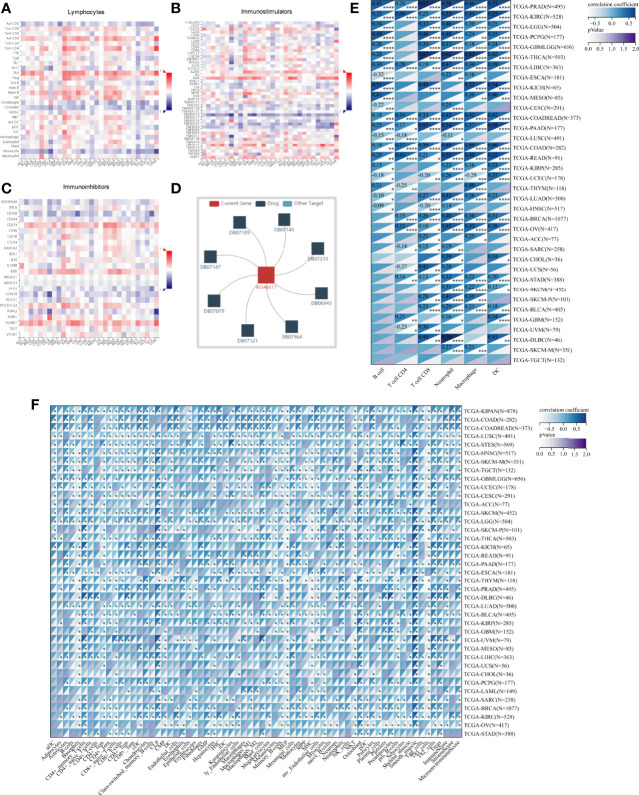
Spearman correlations of the ADAM17 expression with tumor-immune systems in multiple cancer types and targeted drugs. **(A)** Lymphocytes. **(B)** Immunostimulators. **(C)** Immunoinhibitors. **(D)** targeted drugs. **(E)** Correlation between ADAM17 and 6 tumor-related immune cells calculated with TIMER. **(F)** Correlation between ADAM17 and 64 tumor-related immune cells and three Immune cell infiltration scores calculated with the deconvo xCell algorithm. *P < 0.05; **P < 0.005; ***P < 0.001; ****P < 0.0001.

Based on ADAM17 gene expression, we reassessed the invasion scores of aDC, Adipocytes, Astrocytes, B-cells, Basophils, CD4+ memory T-cells, CD4+ naïve T-cells, CD4+ T-cells, CD4+ Tcm, CD4+ Tem, CD8+ naïve T-cells, CD8+ T-cells, CD8+ Tcm, CD8+ Tem, cDC, Chondrocytes, Class-switched_memory_B-cells, CLP, CMP, DC, Endothelial_cells, Eosinophils, Epithelial_cells, Erythrocytes, Fibroblasts, GMP, Hepatocytes, HSC, iDC, Keratinocytes, ly Endothelial cells, Macrophages, M1 Macrophages, M2 Macrophages, Mast cells, Megakaryocytes, Melanocytes, Memory_B-cells, MEP, Mesangial_cells, Monocytes, MPP, MSC, mv Endothelial cells, Myocytes, naïve B-cells, Neurons, Neutrophils, NK cells, NKT, Osteoblast, pDC, Pericytes, Plasma cells, Platelets, Preadipocytes, pro B-cells, Sebocytes, Skeletal_muscle, Smooth_muscle, Tgd_cells, Th1 cells, Th2 cells, Tregs, ImmuneScore, StromaScore, and MicroenvironmentScore in tumor tissues of cancer patients, and found that the expression of ADAM17 was positively related to some lymphocytes (Th2 and Men B) (**Figure 7A**), as well as B cells, CD4 T cells, CD8 T cells, Neutrophils, Macrophages and DC cells (**Figures 7E, F**). It was also positively related to regulatory T cells (Tregs) and M2 Macrophages, which exhibited the inhibition of anti-tumor immunity and promotion of tumor development, respectively (**Figure 7F**). We speculate that ADAM17 is likely to be a novel target for tumor immunotherapy.

In addition, the results uncovered that the expression of ADAM17 was positively correlated with an immunostimulator (IL6R) (**Figure 7B**) and immunoinhibitors (CD274, KDR, TGFBR1) (**Figure 7C**) in most cancer types. However, there was a reverse correlation between the expression of ADAM17 and an immunoinhibitor (TNFRSF14) (**Figure 7B**).

Moreover, since ADAM17 correlated with immune cell infiltration in multiple cancers, drugs targeting ADAM17 from DrugBank database were performed, and results showed that there are eight drugs targeted only for ADAM17 (**Figure 7D**), which all are small molecules (**Table 2**).

**Table 2:** Representative small molecule compounds targeting ADAM17.

DrugBank accession number	Chemical name or compound name	Chemical structure	Chemical formula
DB07189	(1S,3R,6S)-4-oxo-6-{4-[(2-phenylquinolin-4-yl)methoxy]phenyl}-5-azaspiro[2.4]heptane-1-carboxylic acid	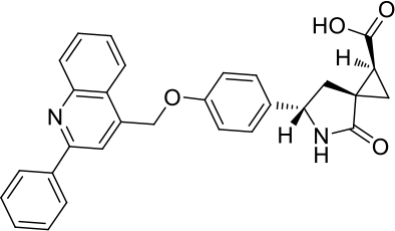	C_29_H_24_N_2_O_4_
DB07145	(2R)-N-hydroxy-2-[(3S)-3-methyl-3-{4-[(2-methylquinolin-4-yl)methoxy]phenyl}-2-oxopyrrolidin-1-yl]propanamide	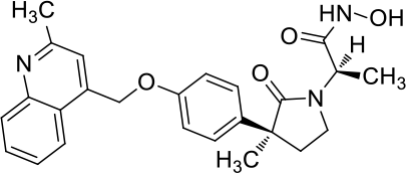	C_25_H_27_N_3_O_4_
DB07233	N-{[4-(but-2-yn-1-yloxy)phenyl]sulfonyl}-5-methyl-D-tryptophan	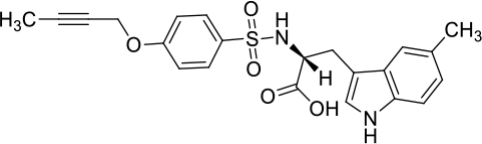	C_22_H_22_N_2_O_5_S
DB06943	(3S)-1-{[4-(but-2-yn-1-yloxy)phenyl]sulfonyl}pyrrolidine-3-thiol	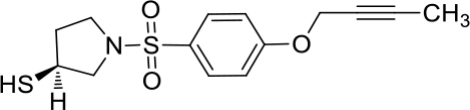	C_14_H_17_NO_3_S_2_
DB07964	(3S)-4-{[4-(but-2-ynyloxy)phenyl]sulfonyl}-N-hydroxy-2,2-dimethylthiomorpholine-3-carboxamide	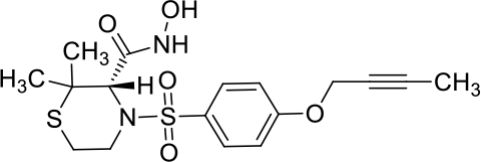	C_17_H_22_N_2_O_5_S_2_
DB07121	4-({4-[(4-aminobut-2-ynyl)oxy]phenyl}sulfonyl)-N-hydroxy-2,2-dimethylthiomorpholine-3-carboxamide	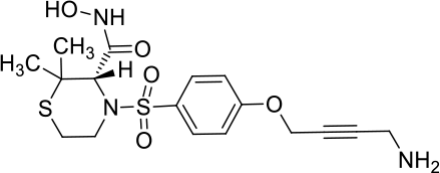	C_17_H_23_N_3_O_5_S_2_
DB07079	3-{[4-(but-2-yn-1-yloxy)phenyl]sulfonyl}propane-1-thiol	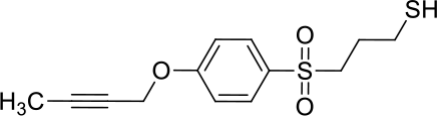	C_13_H_16_O_3_S_2_
DB07147	Methyl(1R,2S)-2-(hydroxycarbamoyl)-1-{4-[(2-methylquinolin-4-yl)methoxy]benzyl}cyclopropanecarboxylate	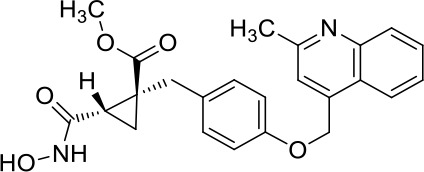	C_24_H_24_N_2_O_5_

## Discussion

As a global public health issue for COVID-19, understanding the expressions and localizations of potential SARS-CoV-2 receptors such as ACE2 and ADAM17 in host tissues may provide insights into prevention or treatments that can reduce the viral infection replication, COVID-19 spread, severity of pathology. It has been shown that ACE2 is implied in viral infection (44–46). Other host receptors like ADAM17 might also function as one of the receptors for viral recognition (47). ADAM17 would closely relate to this viral entry, and the distribution and expression levels of ADAM17 might reflect the susceptibility, viral replication and viral entry. Inhibition of ADAM17 would exert a protective effect on COVID-19 (48). However, the ADAM17 expression in different normal tissues and cancer patients, the impacts of ADAM17 on susceptibility for SARS-CoV-2, and the significance for cancer patients in the COVID-19 outbreaks are unclear.

The homological analysis showed that ADAM17 protein is highly conserved in various species in this study. Its structures and localization prediction indicated the role of ACE2 ectodomain shedding when SARS-CoV-2 attacks, particularly in human airway epithelia (49). ADAM17 is expressed in all normal tissues and upregulated in some tumor tissues, suggesting that all the organs can be potentially infected. In normal lung tissues, ADAM17 mRNA levels increased 30.88-fold more than that in ACE2, and in lung cancer tissues, ADAM17 mRNA levels increased 10-fold more than that of ACE2, suggesting that ADAM17 might play a critical role in SARS-Cov-2 entry in cancer patients *via* lungs, which was supported partially by a systematic review in cancer patients with COVID-19 that lung cancer was more likely to have the risk of COVID-19 when studied the ACE2 expression (43). Moreover, the comparison of ADAM17 expression among normal tissues of the lungs, nasopharynx, and bronchus was also conducted and found to be low at pneumocytes and medium at macrophages. Both protein levels at the nasopharynx and bronchus are medium in respiratory epithelial cells, demonstrating that ADAM17 at respiratory airways might play a critical role in SARS-Cov-2 entry. ADAM17 has also been shown to transform TNF-α precursors into soluble TNF-α, and plays an important role in the processing of many substrates, such as cell adhesion molecule, cytokine growth factor receptor, epidermal growth factor receptor. In addition, ADAM17 can induce diabetes and cardiomyopathy and play an important role in a variety of tumors, such as gastric cancer, breast cancer, prostate cancer, cervical cancer, etc. All these are of great significance to the further study of ADAM17, especially, in cancer patients with SARS-CoV-2.

Furthermore, in cancer patients, higher expression of ADAM17 significantly decreased patient survival in overall survival (OS) in four types of cancers, which was supported by our systematic review results that 7.15% of COVID-19 patients (5,068) had cancer coincidental situations, and the rate of more severe events of COVID-19 patients with malignant cancers (33.33%) presented a higher trend than that for all COVID-19 patients (16.09%) with a significant difference (50).

When the virus enters the body, it will down-regulate the ACE2 level and facilitate the expression of AngII and AT1α receptor in the lung, resulting in the increase of pulmonary capillary permeability and lung injury *via* the activation of the immune system (cytokines and inflammatory factors). In addition, SARS-CoV-2 fatality is attributed to a cytokine storm triggered by excessive pro-inflammatory responses. While attacking the lungs, a large number of cytokines can cause damage to organs outside the lungs, such as the heart, kidney, liver, and other organs. A large number of plasma cytokines and chemokines (i.e., CCL2, CXCL8, TNFα, etc) were found to be accumulated in patients with SARS-CoV-2 (51). In addition to acting as a viral binding receptor, ACE2 is also involved in modulating immune processes, leading to cytokine storms and exacerbation of pneumonia (52). ADAM17 may be the master molecule involved in regulating IL-6 class switching, and through this, it can control inflammatory response to viral antigenic stimuli (53). We thus focus on analyzing the expressions of ADAM17 in normal heart tissues because the cytokine can easily increase the severity in younger people and eventually lead to death (54, 55). The expressions in normal heart tissues of ADAM17 protein were medium, whereas those in normal heart tissues of ACE2 protein were under medium or lower, localizing in both membrane and cytoplasm. The mRNA levels of ADAM17 are increased 1.28-fold than that of ACE2 in heart muscles. ADAM17, as a type I multi-domain transmembrane protein, has a similar function to ACE2 and contributes to the hydrolysis and shedding of ACE2, while an increase in shedding of ACE2 can aggravate viral infection. Therefore, we speculate that ADAM17 might play an important role in cytokine storms. Indeed, compared to normal values, cytokine factors, including IL-6, were higher in severe patients with COVID-19 (56, 57). Thus, ADAM17 should be considered a potential target for drug discovery that regulates host reactivity to viral infection and prevents fatal outcomes (58).

Moreover, drugs targeting ADAM17 from the DrugBank database showed eight small molecule compounds targeted only for ADAM17, demonstrating the therapeutic significance of targeting ADAM17 for both the prevention of tumor progression and SARS-CoV-2 attacking in immune implications.

## Conclusion

In summary, our analysis uncovered that ADAM17 is expressed in both normal (especially in the lung) and tumor tissues and is highly expressed in several tumor samples. High expression of ADAM17 significantly reduces the prognosis of patients with malignant tumors. These results suggest that ADAM17 may promote coronavirus infection in patients with malignant tumors. Lung tissue cell analysis showed that ADAM17 protein was highly expressed in respiratory epithelial cells of the nasopharynx and bronchus, suggesting that viral infection may be mainly distributed in respiratory epithelial cells. Eight drugs were targeted only for ADAM17, demonstrating the immune implications of targeting ADAM17 to prevent tumor progression and SARS-CoV-2 attack. These drugs that might be useful for targeting ADAM17 and SARS-CoV-2 in addition to enhancement of respiratory tract immune defense.

## Data Availability Statement

The original contributions presented in the study are included in the article/**Supplementary Material**. Further inquiries can be directed to the corresponding author.

## Ethics Statement

The study was approved by the Ethical Committee of Southwest Medical University. The patients/participants provided their written informed consent to participate in this study.

## Author Contributions

JF conceived and coordinated the study. JF, JH, DL, HD, ZM, BS, KW, JC, and JiF conducted experiments and analyzed and interpreted data. LZ provided pathology expertise. KW and KSB edited the manuscript. JuF wrote and edited the manuscript. All authors agreed that the manuscript should be submitted to the Journal.

## Funding

This work was supported in part by the National Natural Science Foundation of China (grant nos. 82073263, 81672887, and 30371493), the Research Foundation of Luzhou City (grant no. 2021-SYF-37), the Foundation of Southwest Medical University (grant nos. 2021ZKMS004, 2021ZKQN109), and the Translational Medicine Foundation of Southwest Medical University (grant no. 00031476).

## Conflict of Interest Statement

The authors declare that the research was conducted in the absence of any commercial or financial relationships that could be construed as a potential conflict of interest.

## Publisher’s Note

All claims expressed in this article are solely those of the authors and do not necessarily represent those of their affiliated organizations, or those of the publisher, the editors and the reviewers. Any product that may be evaluated in this article, or claim that may be made by its manufacturer, is not guaranteed or endorsed by the publisher.

## Abbreviations

SARS-Cov-2, Severe acute respiratory syndrome coronavirus 2; COVID-19, Coronavirus Disease 2019; ADAM17, A disintegrin and metalloproteinase domain 17; ACE2, Angiotensin-Converting Enzyme 2; HPA, Human Protein Atlas; NX, Normalized expression; IHC, Immunohistochemistry; GEPIA, The Gene Expression Profiling Interactive Analysis; TCGA, The Cancer Genome Atlas.

## References

[1] Khan RJ Jha RK Amera GM Jain M Singh E Pathak A. Targeting Sars-Cov-2: A Systematic Drug Repurposing Approach to Identify Promising Inhibitors Against 3c-Like Proteinase and 2'-O-Ribose Methyltransferase. J biomol struct dynam (2021) 39(8):2679–92. doi: 10.1080/07391102.2020.1753577 PMC7189412 32266873

[2] Rossi GA Sacco O Mancino E Cristiani L Midulla F. Differences and Similarities Between Sars-Cov and Sars-Cov-2: Spike Receptor-Binding Domain Recognition and Host Cell Infection With Support of Cellular Serine Proteases. Infection (2020) 48(5):665–9. doi: 10.1007/s15010-020-01486-5 PMC7393809 32737833

[3] V'Kovski P Kratzel A Steiner S Stalder H Thiel V. Coronavirus Biology and Replication: Implications for Sars-Cov-2. Nat Rev Microbiol (2021) 19(3):155–70. doi: 10.1038/s41579-020-00468-6 PMC7592455 33116300

[4] Hasan A Paray BA Hussain A Qadir FA Attar F Aziz FM. A Review on the Cleavage Priming of the Spike Protein on Coronavirus by Angiotensin-Converting Enzyme-2 and Furin. J biomol struct dynam (2021) 39(8):3025–33. doi: 10.1080/07391102.2020.1754293 PMC7189411 32274964

[5] Ibrahim IM Abdelmalek DH Elshahat ME Elfiky AA. Covid-19 Spike-Host Cell Receptor Grp78 Binding Site Prediction. J infect (2020) 80(5):554–62. doi: 10.1016/j.jinf.2020.02.026 PMC7102553 32169481

[6] Li D Liu X Zhang L He J Chen X Liu S. Covid-19 Disease and Malignant Cancers: The Impact for the Furin Gene Expression in Susceptibility to Sars-Cov-2. Int J Biol Sci (2021) 17(14):3954–67. doi: 10.7150/ijbs.63072 PMC8495395 34671211

[7] Ziegler CGK Allon SJ Nyquist SK Mbano IM Miao VN Tzouanas CN. Sars-Cov-2 Receptor Ace2 Is an Interferon-Stimulated Gene in Human Airway Epithelial Cells and Is Detected in Specific Cell Subsets Across Tissues. Cell (2020) 181(5):1016–35.e19. doi: 10.1016/j.cell.2020.04.035 32413319 PMC7252096

[8] Abassi Z Higazi AAR Kinaneh S Armaly Z Skorecki K Heyman SN. Ace2, Covid-19 Infection, Inflammation, and Coagulopathy: Missing Pieces in the Puzzle. Front Physiol (2020) 11:574753. doi: 10.3389/fphys.2020.574753 33123031 PMC7573220

[9] Mukhopadhyay D AlSawaftah N Husseini GA. Identification of Novel Micrornas as Promising Therapeutics for Sars-Cov-2 by Regulating the Egfr-Adam17 Axis: An in Silico Analysis. ACS Pharmacol Trans Sci (2021) 4(1):396–9. doi: 10.1021/acsptsci.0c00199 PMC7887851 33615189

[10] Dobert JP Cabron AS Arnold P Pavlenko E Rose-John S Zunke F. Functional Characterization of Colon-Cancer-Associated Variants in Adam17 Affecting the Catalytic Domain. Biomedicines (2020) 8(11):463. doi: 10.3390/biomedicines8110463 PMC7692748 33143292

[11] Healy EF Lilic M. A Model for Covid-19-Induced Dysregulation of Ace2 Shedding by Adam17. Biochem Biophys Res Commun (2021) 573:158–63. doi: 10.1016/j.bbrc.2021.08.040 PMC8364680 34416436

[12] Xu K Dai L Gao GF. Humoral and Cellular Immunity and the Safety of Covid-19 Vaccines: A Summary of Data Published by 21 May 2021. Int Immunol (2021) 33(10):529–40. doi: 10.1093/intimm/dxab061 PMC8499872 34491327

[13] Alsayb MA Alsamiri ADD Makhdoom HQ Alwasaidi T Osman HM Mahallawi WH. Prolonged Humoral and Cellular Immunity in Covid-19-Recovered Patients. Saudi J Biol Sci (2021) 28(7):4010–5. doi: 10.1016/j.sjbs.2021.04.008 PMC8040318 33867805

[14] Hiam-Galvez KJ Allen BM Spitzer MH. Systemic Immunity in Cancer. Nat Rev Cancer (2021) 21(6):345–59. doi: 10.1038/s41568-021-00347-z PMC8034277 33837297

[15] Liang W Guan W Chen R Wang W Li J Xu K. Cancer Patients in Sars-Cov-2 Infection: A Nationwide Analysis in China. Lancet Oncol (2020) 21(3):335–7. doi: 10.1016/S1470-2045(20)30096-6 PMC7159000 32066541

[16] Yu J Ouyang W Chua MLK Xie C. Sars-Cov-2 Transmission in Patients With Cancer at a Tertiary Care Hospital in Wuhan, China. JAMA Oncol (2020) 6(7):1108–10. doi: 10.1001/jamaoncol.2020.0980 PMC7097836 32211820

[17] Sinnathamby G Zerfass J Hafner J Block P Nickens Z Hobeika A. Adam Metallopeptidase Domain 17 (Adam17) Is Naturally Processed Through Major Histocompatibility Complex (Mhc) Class I Molecules and Is a Potential Immunotherapeutic Target in Breast, Ovarian and Prostate Cancers. Clin Exp Immunol (2011) 163(3):324–32. doi: 10.1111/j.1365-2249.2010.04298.x PMC3048615 21175594

[18] Uhlen M Fagerberg L Hallstrom BM Lindskog C Oksvold P Mardinoglu A. Proteomics. Tissue-Based Map of the Human Proteome. Science (2015) 347(6220):1260419. doi: 10.1126/science.1260419 25613900

[19] Uhlen M Oksvold P Fagerberg L Lundberg E Jonasson K Forsberg M. Towards a Knowledge-Based Human Protein Atlas. Nat Biotechnol (2010) 28(12):1248–50. doi: 10.1038/nbt1210-1248 21139605

[20] Thul PJ Akesson L Wiking M Mahdessian D Geladaki A Ait Blal H. A Subcellular Map of the Human Proteome. Science (2017) 356(6340):eaal3321. doi: 10.1126/science.aal3321 28495876

[21] Uhlen M Zhang C Lee S Sjostedt E Fagerberg L Bidkhori G. A Pathology Atlas of the Human Cancer Transcriptome. Science (2017) 357(6352):eaan2507. doi: 10.1126/science.aan2507 28818916

[22] Tang Z Li C Kang B Gao G Li C Zhang Z. Gepia: A Web Server for Cancer and Normal Gene Expression Profiling and Interactive Analyses. Nucleic Acids Res (2017) 45(W1):W98–W102. doi: 10.1093/nar/gkx247 28407145 PMC5570223

[23] Fu J Zhou B Zhang L Balaji KS Wei C Liu X. Expressions and Significances of the Angiotensin-Converting Enzyme 2 Gene, the Receptor of Sars-Cov-2 for Covid-19. Mol Biol Rep (2020) 47(6):4383–92. doi: 10.1007/s11033-020-05478-4 PMC7224351 32410141

[24] Ru B Wong CN Tong Y Zhong JY Zhong SSW Wu WC. Tisidb: An Integrated Repository Portal for Tumor-Immune System Interactions. Bioinformatics (2019) 35(20):4200–2. doi: 10.1093/bioinformatics/btz210 30903160

[25] Cheng J Zhou J Fu S Fu J Zhou B Chen H. Prostate Adenocarcinoma and Covid-19: The Possible Impacts of Tmprss2 Expressions in Susceptibility to Sars-Cov-2. J Cell Mol Med (2021) 25(8):4157–65. doi: 10.1111/jcmm.16385 PMC8013364 33609069

[26] Berglund L Bjorling E Oksvold P Fagerberg L Asplund A Szigyarto CA. A Genecentric Human Protein Atlas for Expression Profiles Based on Antibodies. Mol Cell Proteomics MCP (2008) 7(10):2019–27. doi: 10.1074/mcp.R800013-MCP200 18669619

[27] Zhang L Yang M Gan L He T Xiao X Stewart MD. Dlx4 Upregulates Twist and Enhances Tumor Migration, Invasion and Metastasis. Int J Biol Sci (2012) 8(8):1178–87. doi: 10.7150/ijbs.4458 PMC3477687 23091415

[28] Fu J Zhang L He T Xiao X Liu X Wang L. Twist Represses Estrogen Receptor-Alpha Expression by Recruiting the Nurd Protein Complex in Breast Cancer Cells. Int J Biol Sci (2012) 8(4):522–32. doi: 10.7150/ijbs.4164 PMC3314193 22457607

[29] Zhang L Wei C Li D He J Liu S Deng H. Covid-19 Receptor and Malignant Cancers: Association of Ctsl Expression With Susceptibility to Sars-Cov-2. Int J Biol Sci (2022) 18(6):2362–71. doi: 10.7150/ijbs.70172 PMC8990473 35414771

[30] Thorsson V Gibbs DL Brown SD Wolf D Bortone DS Ou Yang TH. The Immune Landscape of Cancer. Immunity (2018) 48(4):812–30.e14. doi: 10.1016/j.immuni.2018.03.023 29628290 PMC5982584

[31] Li T Fan J Wang B Traugh N Chen Q Liu JS. Timer: A Web Server for Comprehensive Analysis of Tumor-Infiltrating Immune Cells. Cancer Res (2017) 77(21):e108–e10. doi: 10.1158/0008-5472.CAN-17-0307 PMC6042652 29092952

[32] Aran D Hu Z Butte AJ. Xcell: Digitally Portraying the Tissue Cellular Heterogeneity Landscape. Genome Biol (2017) 18(1):220. doi: 10.1186/s13059-017-1349-1 29141660 PMC5688663

[33] Zeng D Ye Z Shen R Yu G Wu J Xiong Y. Iobr: Multi-Omics Immuno-Oncology Biological Research to Decode Tumor Microenvironment and Signatures. Front Immunol (2021) 12:687975. doi: 10.3389/fimmu.2021.687975 34276676 PMC8283787

[34] Yan R Zhang Y Li Y Xia L Guo Y Zhou Q. Structural Basis for the Recognition of Sars-Cov-2 by Full-Length Human Ace2. Science (2020) 367(6485):1444–8. doi: 10.1126/science.abb2762 PMC7164635 32132184

[35] Takeda S. Adam and Adamts Family Proteins and Snake Venom Metalloproteinases: A Structural Overview. Toxins (2016) 8(5):155. doi: 10.3390/toxins8050155 PMC4885070 27196928

[36] Fox JW Serrano SM. Structural Considerations of the Snake Venom Metalloproteinases, Key Members of the M12 Reprolysin Family of Metalloproteinases. Toxicon (2005) 45(8):969–85. doi: 10.1016/j.toxicon.2005.02.012 15922769

[37] Van Wart HE Birkedal-Hansen H. The Cysteine Switch: A Principle of Regulation of Metalloproteinase Activity With Potential Applicability to the Entire Matrix Metalloproteinase Gene Family. Proc Natl Acad Sci USA (1990) 87(14):5578–82. doi: 10.1073/pnas.87.14.5578 PMC54368 2164689

[38] McLane MA Sanchez EE Wong A Paquette-Straub C Perez JC. Disintegrins. Curr Drug Targets Cardiovasc Haematol Disord (2004) 4(4):327–55. doi: 10.2174/1568006043335880 15578957

[39] Hendrickson WA Teeter MM. Structure of the Hydrophobic Protein Crambin Determined Directly From the Anomalous Scattering of Sulphur. Nature (1981) 290(5802):107–13. doi: 10.1038/290107a0 PMC5536114 28769131

[40] Heurich A Hofmann-Winkler H Gierer S Liepold T Jahn O Pohlmann S. Tmprss2 and Adam17 Cleave Ace2 Differentially and Only Proteolysis by Tmprss2 Augments Entry Driven by the Severe Acute Respiratory Syndrome Coronavirus Spike Protein. J Virol (2014) 88(2):1293–307. doi: 10.1128/JVI.02202-13 PMC3911672 24227843

[41] Kuba K Imai Y Rao S Gao H Guo F Guan B. A Crucial Role of Angiotensin Converting Enzyme 2 (Ace2) in Sars Coronavirus-Induced Lung Injury. Nat Med (2005) 11(8):875–9. doi: 10.1038/nm1267 PMC7095783 16007097

[42] Leow MKS. Clarifying the Controversial Risk-Benefit Profile of Soluble Ace2 in Covid-19. Crit Care (2020) 24(1):396. doi: 10.1186/s13054-020-03097-w 32631373 PMC7338146

[43] Ren P Gong C Ma S. Evaluation of Covid-19 Based on Ace2 Expression in Normal and Cancer Patients. Open Med (2020) 15(1):613–22. doi: 10.1515/med-2020-0208 PMC7712168 33336018

[44] Hikmet F Mear L Edvinsson A Micke P Uhlen M Lindskog C. The Protein Expression Profile of Ace2 in Human Tissues. Mol Syst Biol (2020) 16(7):e9610. doi: 10.15252/msb.20209610 32715618 PMC7383091

[45] Lan J Ge J Yu J Shan S Zhou H Fan S. Structure of the Sars-Cov-2 Spike Receptor-Binding Domain Bound to the Ace2 Receptor. Nature (2020) 581(7807):215–20. doi: 10.1038/s41586-020-2180-5 32225176

[46] Shang J Ye G Shi K Wan Y Luo C Aihara H. Structural Basis of Receptor Recognition by Sars-Cov-2. Nature (2020) 581(7807):221–4. doi: 10.1038/s41586-020-2179-y PMC7328981 32225175

[47] Schreiber B Patel A Verma A. Shedding Light on Covid-19: Adam17 the Missing Link? Am J Ther (2020) 28(3):e358–60. doi: 10.1097/MJT.0000000000001226 32769398

[48] Palau V Riera M Soler MJ. Adam17 Inhibition May Exert a Protective Effect on Covid-19. Nephrol dialysis Transplant Off Publ Eur Dialysis Transplant Assoc - Eur Renal Assoc (2020) 35(6):1071–2. doi: 10.1093/ndt/gfaa093 PMC7184459 32291449

[49] Jia HP Look DC Tan P Shi L Hickey M Gakhar L. Ectodomain Shedding of Angiotensin Converting Enzyme 2 in Human Airway Epithelia. Am J Physiol Lung Cell Mol Physiol (2009) 297(1):L84–96. doi: 10.1152/ajplung.00071.2009 PMC2711803 19411314

[50] Fu J Wei C He J Zhang L Zhou J Balaji KS. Evaluation and Characterization of Hspa5 (Grp78) Expression Profiles in Normal Individuals and Cancer Patients With Covid-19. Int J Biol Sci (2021) 17(3):897–910. doi: 10.7150/ijbs.54055 33767597 PMC7975696

[51] Wang J Kaplan N Wysocki J Yang W Lu K Peng H. The Ace2-Deficient Mouse: A Model for a Cytokine Storm-Driven Inflammation. FASEB J Off Publ Fed Am Soc Exp Biol (2020) 34(8):10505–15. doi: 10.1096/fj.202001020R PMC7323146 32725927

[52] Chen L Liu Y Wu J Deng C Tan J Liu H. Lung Adenocarcinoma Patients Have Higher Risk of SARS-CoV-2 Infection. Aging (Albany NY) (2021) 13(2):1620–32. doi: 10.18632/aging.202375 PMC7880402 33429366

[53] Gomez MI Sokol SH Muir AB Soong G Bastien J Prince AS. Bacterial Induction of Tnf-Alpha Converting Enzyme Expression and Il-6 Receptor Alpha Shedding Regulates Airway Inflammatory Signaling. J Immunol (2005) 175(3):1930–6. doi: 10.4049/jimmunol.175.3.1930 16034137

[54] Ben Moftah M Eswayah A. Intricate Relationship Between Sars-Cov-2-Induced Shedding and Cytokine Storm Generation: A Signaling Inflammatory Pathway Augmenting Covid-19. Health Sci Rev (2022) 2:100011. doi: 10.1016/j.hsr.2021.100011 PMC8734057 35013738

[55] Karki R Sharma BR Tuladhar S Williams EP Zalduondo L Samir P. Synergism of Tnf-Alpha and Ifn-Gamma Triggers Inflammatory Cell Death, Tissue Damage, and Mortality in Sars-Cov-2 Infection and Cytokine Shock Syndromes. Cell (2021) 184(1):149–68.e17. doi: 10.1016/j.cell.2020.11.025 33278357 PMC7674074

[56] Wan S Yi Q Fan S Lv J Zhang X Guo L. Relationships Among Lymphocyte Subsets, Cytokines, and the Pulmonary Inflammation Index in Coronavirus (Covid-19) Infected Patients. Br J haematol (2020) 189(3):428–37. doi: 10.1111/bjh.16659 PMC7262036 32297671

[57] Xu B Fan CY Wang AL Zou YL Yu YH He C. Suppressed T Cell-Mediated Immunity in Patients With Covid-19: A Clinical Retrospective Study in Wuhan, China. J infect (2020) 81(1):e51–60. doi: 10.1016/j.jinf.2020.04.012 PMC7166040 32315725

[58] Mahmud-Al-Rafat A Majumder A Taufiqur Rahman KM Mahedi Hasan AM Didarul Islam KM Taylor-Robinson AW. Decoding the Enigma of Antiviral Crisis: Does One Target Molecule Regulate All? Cytokine (2019) 115:13–23. doi: 10.1016/j.cyto.2018.12.008 30616034 PMC7129598

