# Supplementary material for: The Correlation Between Immune Invasion and SARS-COV-2 Entry Protein ADAM17 in Cancer Patients by Bioinformatic Analysis
Source: Front Immunol. 2022 Jun 3;13:923516. doi: 10.3389/fimmu.2022.923516 (PMC9203860; doi:10.3389/fimmu.2022.923516)
Supplement: Supplementary file 1 [file Presentation_1.pptx]

## Slide 1
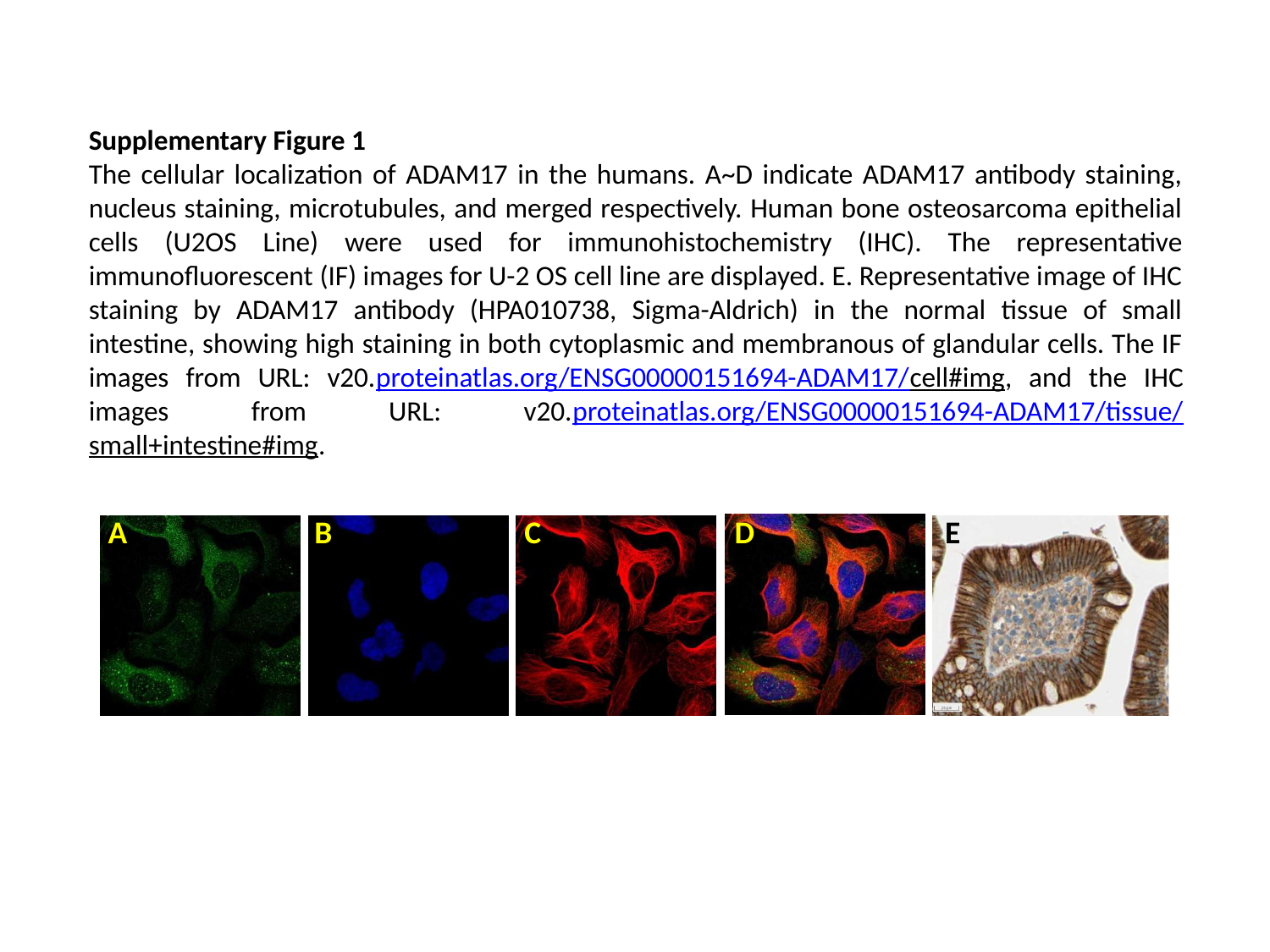

Supplementary Figure 1
The cellular localization of ADAM17 in the humans. A~D indicate ADAM17 antibody staining, nucleus staining, microtubules, and merged respectively. Human bone osteosarcoma epithelial cells (U2OS Line) were used for immunohistochemistry (IHC). The representative immunofluorescent (IF) images for U-2 OS cell line are displayed. E. Representative image of IHC staining by ADAM17 antibody (HPA010738, Sigma-Aldrich) in the normal tissue of small intestine, showing high staining in both cytoplasmic and membranous of glandular cells. The IF images from URL: v20.proteinatlas.org/ENSG00000151694-ADAM17/cell#img, and the IHC images from URL: v20.proteinatlas.org/ENSG00000151694-ADAM17/tissue/small+intestine#img.
A
B
C
D
E

## Slide 2
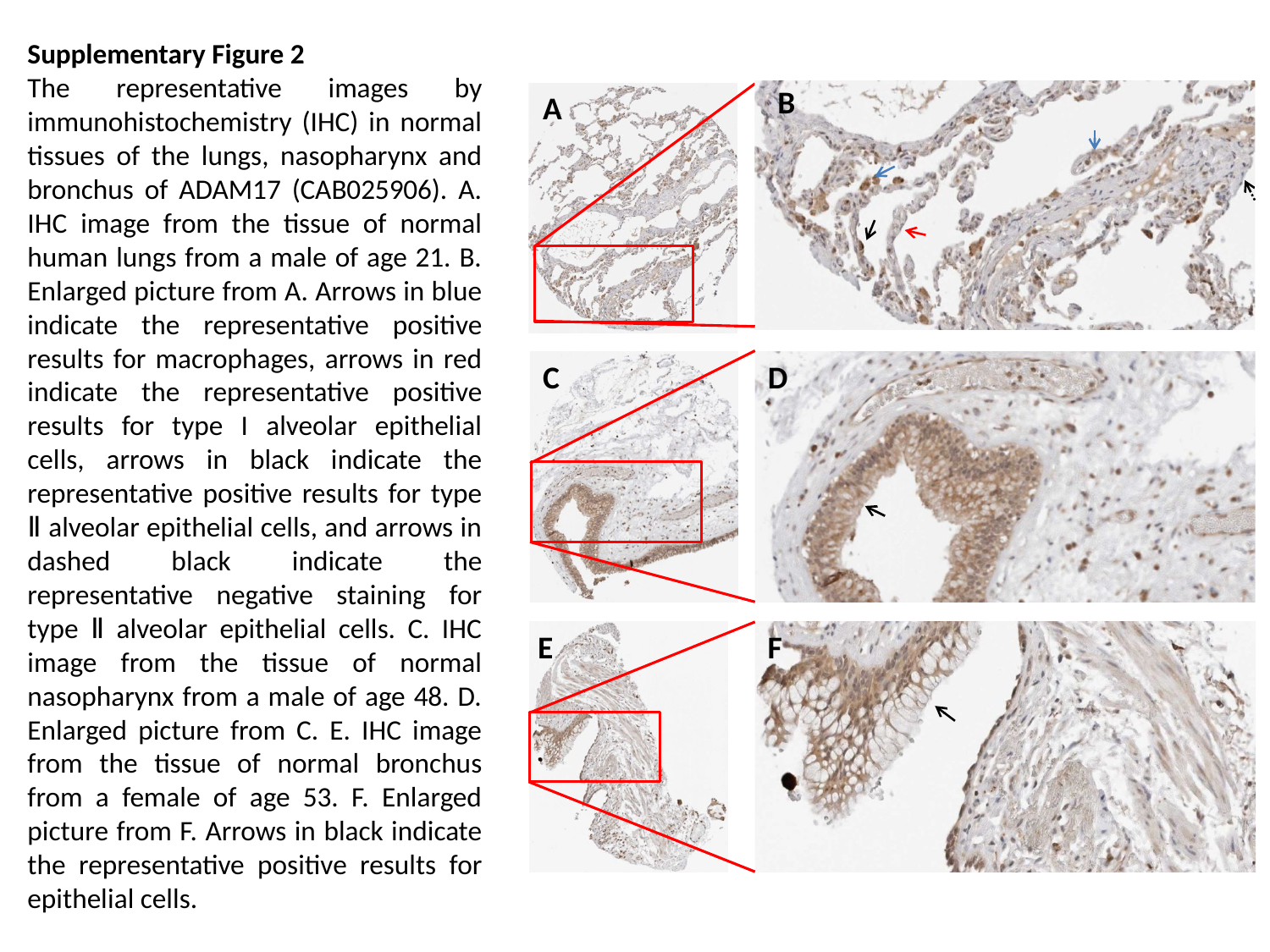

Supplementary Figure 2
The representative images by immunohistochemistry (IHC) in normal tissues of the lungs, nasopharynx and bronchus of ADAM17 (CAB025906). A. IHC image from the tissue of normal human lungs from a male of age 21. B. Enlarged picture from A. Arrows in blue indicate the representative positive results for macrophages, arrows in red indicate the representative positive results for type I alveolar epithelial cells, arrows in black indicate the representative positive results for type Ⅱ alveolar epithelial cells, and arrows in dashed black indicate the representative negative staining for type Ⅱ alveolar epithelial cells. C. IHC image from the tissue of normal nasopharynx from a male of age 48. D. Enlarged picture from C. E. IHC image from the tissue of normal bronchus from a female of age 53. F. Enlarged picture from F. Arrows in black indicate the representative positive results for epithelial cells.
B
A
D
C
F
E

## Slide 3
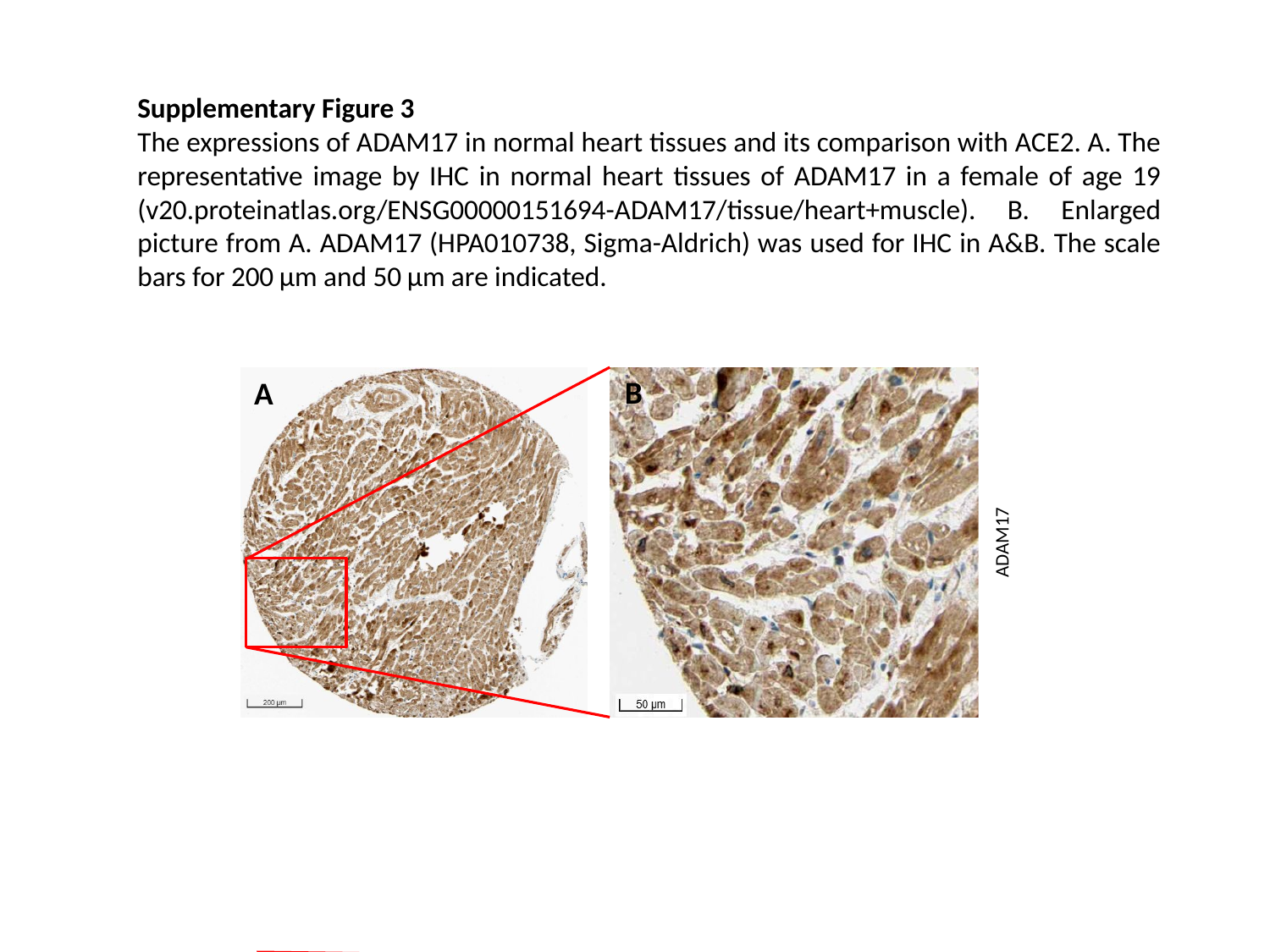

Supplementary Figure 3
The expressions of ADAM17 in normal heart tissues and its comparison with ACE2. A. The representative image by IHC in normal heart tissues of ADAM17 in a female of age 19 (v20.proteinatlas.org/ENSG00000151694-ADAM17/tissue/heart+muscle). B. Enlarged picture from A. ADAM17 (HPA010738, Sigma-Aldrich) was used for IHC in A&B. The scale bars for 200 µm and 50 µm are indicated.
B
A
ADAM17

## Slide 4
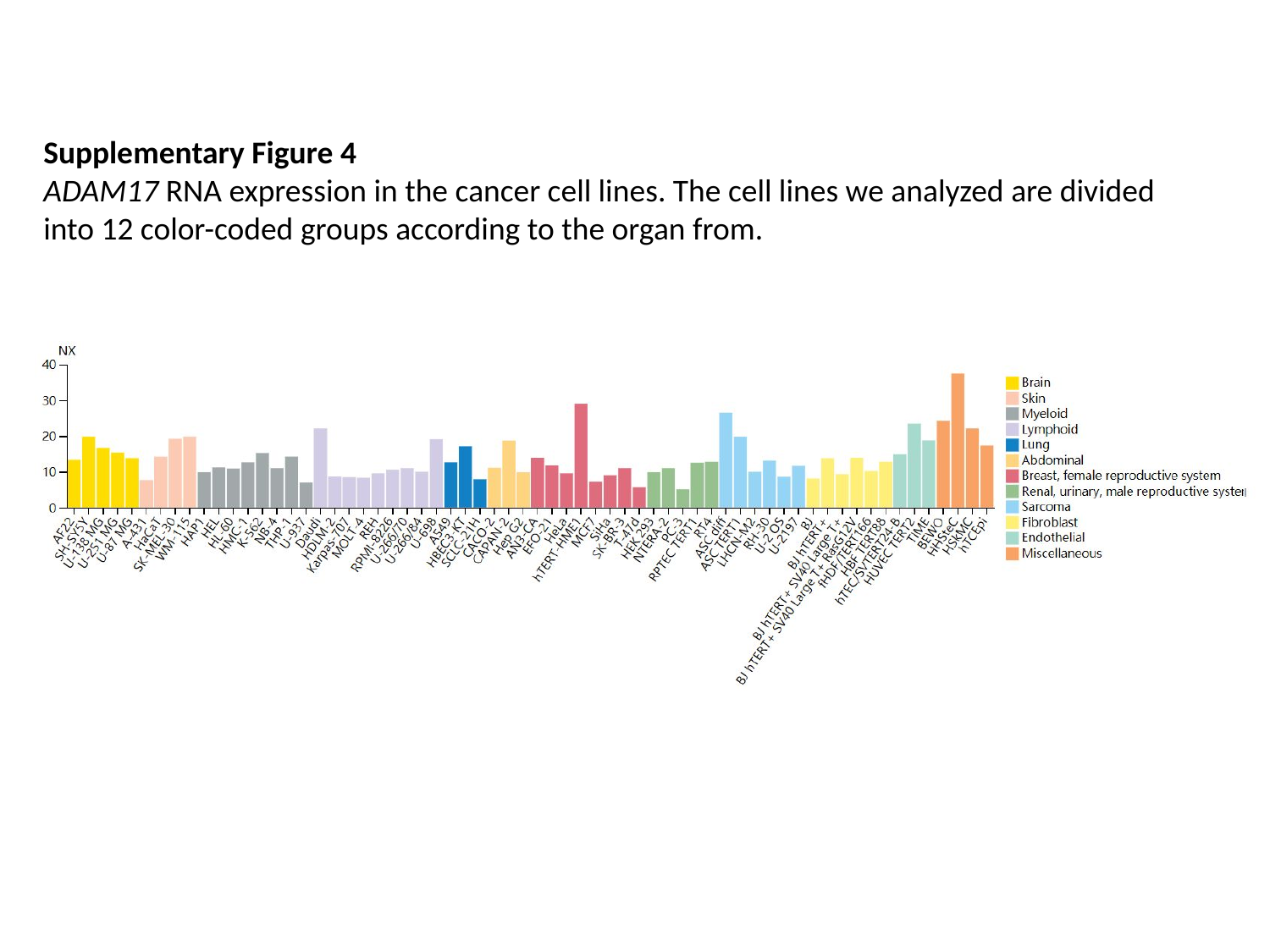

Supplementary Figure 4
ADAM17 RNA expression in the cancer cell lines. The cell lines we analyzed are divided into 12 color-coded groups according to the organ from.
